# The evolutionary genomics of meiotic drive

**DOI:** 10.1093/molbev/msag020

**Published:** 2026-01-23

**Authors:** Daven C Presgraves, R Kelly Dawe, Kelly A Dyer, Lila Fishman, Soumitra A Bhide, Sasha L Bradshaw, Meghan J Brady, Alejandro Burga, Cécile Courret, Brandon L Fagen, Ana Beatriz Stein Machado Ferretti, Reka K Kelemen, Jun Kitano, Yiran Liu, Emiliano Martí, Theresa Erlenbach, Josephine A Reinhardt, Laura Ross, Jan-Niklas Runge, Callie M Swanepoel, Beatriz Vicoso, Aaron A Vogan, Anna K Lindholm, Amanda M Larracuente, Robert L Unckless

**Affiliations:** Department of Biology, University of Rochester, Rochester, NY, USA; Department of Genetics, University of Georgia, Athens, GA, USA; Department of Genetics, University of Georgia, Athens, GA, USA; Division of Biological and Biomedical Sciences, University of Montana, Missoula, MT, USA; School of BioSciences, The University of Melbourne, Melbourne 3010, Victoria, Australia; Genetics, Evolution, and Environment, University College London, London, UK; Department of Genetics, University of Georgia, Athens, GA, USA; Institute of Molecular Biotechnology of the Austrian Academy of Sciences (IMBA), Vienna BioCenter (VBC), Dr. Bohr-Gasse 3, Vienna 1030, Austria; Laboratoire Evolution, Génome, Comportement et Ecologie, UMR UPSaclay, CNRS 9191, IRD 247, Gif-sur-Yvette, France; Stowers Institute for Medical Research, Kansas City, MO, USA; Departamento de Biologia Geral e Aplicada, UNESP - Univ Estadual Paulista, Instituto de Biociências/IB, Rio Claro, São Paulo, Brazil; Institute of Science and Technology Austria, Klosterneuberg 3400, Austria; Ecological Genetics Laboratory, National Institute of Genetics, Mishima, Shizuoka, Japan; Center for Bioinformatics, Center for Life Sciences, School of Life Sciences, Peking University, Beijing, China; Department of Biology, University of Rochester, Rochester, NY, USA; Department of Genetics, University of Georgia, Athens, GA, USA; Department of Biology, The State University of New York College at Geneseo, Geneseo, NY, USA; School of Biological Sciences, Institute of Ecology and Evolution, The University of Edinburgh, Edinburgh, UK; CNRS GMGM UMR 7156, Université de Strasbourg, Strasbourg, France; Department of Human Genetics, University of Utah, Salt Lake City, UT, USA; Institute of Science and Technology Austria, Klosterneuberg 3400, Austria; Department of Organismal Biology, Uppsala University, Uppsala, Sweden; Department of Evolutionary Biology and Environmental Studies, University of Zurich, Zurich, Switzerland; Department of Biology, University of Rochester, Rochester, NY, USA; Department of Molecular Biosciences, University of Kansas, Lawrence, KS, USA

**Keywords:** meiotic drive, genome evolution, selfish genetic elements

## Abstract

Meiotic drivers are selfish genetic elements that gain transmission advantages by distorting equal, Mendelian segregation. For decades, biologists have considered meiotic drivers as interesting, albeit esoteric, case studies. It is now clear, however, that meiotic drive is more common and phylogenetically widespread than previously supposed. Indeed, intensive study of a few well-known cases has begun to reveal the evolutionary genomic consequences of meiotic drive. We argue here that many features of genome evolution, content, and organization that are seemingly inexplicable by organismal adaptation or nearly neutral processes are instead best accounted for by recurrent histories of meiotic drive. We review how meiotic drive can affect the evolution of sequences, gene copy numbers, genes with functions in meiosis and gametogenesis, signatures of “selection,” chromosome rearrangements, and karyotype evolution. We also explore the interactions of meiotic drive elements with other classes of selfish genetic elements, including satellite DNAs, transposable elements, and with the endogenous host genes involved in drive suppression. Finally, we argue that some aspects of drive-mediated genome evolution are now sufficiently well established that we might reverse the direction of discovery—rather than ask how drive affects genome evolution, we can use genome data to discover new putative drive elements.

## Introduction

The genomes of all eukaryotes host diverse communities of evolutionarily “selfish” genetic elements (SGEs)—DNA sequences that gain transmission advantages but confer no fitness benefits to (or, worse, impose costs on) their bearers (Werren et al. 1988; Burt and Trivers 2006). To do so, SGEs tend to deploy one of two broad strategies. Transposable elements (TEs) *over-replicate* relative to host genomes by, e.g. producing and mobilizing excess copies that insert into naïve, “uninfected” chromosomes. Meiotic drive elements secure more than their fair (50%) share of transmission by incapacitating or outcompeting gametes that lack them. While SGEs provide important proofs-of-principle—they, for instance, legitimize a selfish gene (or gene's-eye) view of evolution (Oestergren and Prakken 1946; Dawkins 1976; Ågren 2021)—questions on their biological significance have long stirred debate. Historically, SGEs have been considered rare curiosities. The reason is that SGE phenotypes are typically transient and/or cryptic, a problem not dissimilar to the more general challenge of observing natural selection in action. Today, of course, we have many methods to detect ongoing and/or past natural selection, including those that rely on signatures of selection in genome sequences. We suggest here that genomes likewise bear signatures of ongoing and/or past SGE activity and evolution.

The case is hardly controversial for TEs, where the story is the stuff of textbooks. It begins with Barbara McClintock's discovery of the unorthodox genetics of an autonomous mobile factor, termed *Activator* (*Ac*), which induces chromosome breakage and mutations in maize by activating a non-autonomous mobile factor, termed *Dissociation* (*Ds*; reviewed in Fedoroff (1989)). Her findings attracted scant attention for ∼15 years, until similar mobile factors were found to cause similar genetic instabilities in bacteria and in *Drosophila*, thus establishing TEs as something more than peculiarities of maize. The molecular characterization of TEs quickly revealed strong sequence and/or structural themes: They tend to be small (<10 kb; but see Arkhipova and Yushenova (2019)); encode proteins not endemic to host genomes (e.g. reverse transcriptase, transposase); often possess short terminal repeat sequences; sometimes spawn non-autonomous derivatives; frequently give rise to degenerate copies; and, of course, exist as dispersed, multicopy sequences. Indeed, TEs are sufficiently stereotypical that they are now routinely discovered and annotated in silico using standard software. This pivot from the laborious, piecemeal, forward genetic characterization to the *en masse* reverse characterization of TE sequences in genomes has been monumental. We now know, for instance, that TEs comprise the single largest fraction of many eukaryotic genome sequences—hardly a sideshow—and, while most are parasitic, others have become coopted for essential host functions (reviewed in Cosby et al. (2019)). The ubiquity of TEs is now such a commonplace, that it is easy to forget that just ∼45 years ago the classic selfish DNA papers of Doolittle and Sapienza (1980) and Orgel and Crick (1980) were controversial: Upsetting the adaptationist status quo of the time, they argued that huge portions of eukaryotic genomes exist in service of selfish repetitive elements rather than organismal functions.

The story for meiotic drive is less clearly developed, and our understanding of its impact on genomes has lagged for several reasons. For one, drive does not always present overt phenotypes. It is no coincidence that the best-known drive cases are those incidentally associated with mutations with visible phenotypes, biased sex ratios, or strong cytological phenotypes (see below). Next, while drive phenomena were first described a century ago (Morgan et al. 1925; Dobrovolskaia-Zavadskaia and Kobozieff 1927; Gershenson 1928; Chesley and Dunn 1936; Rhoades 1942; Hiraizumi and Crow 1960), the success of forward genetics in determining the molecular identities of drive sequences has been slow. Finally, while drive has been discovered in virtually every major eukaryotic system to come under laboratory scrutiny—plants, fungi, insects, fish, and mammals—the genetics of drive has been slower to converge on common sequence and/or structural themes. Drive elements, it seems, act via a wider range of sequences, structures, and molecular mechanisms than TEs. Despite these limitations, we argue here that studies of drive have approached a critical mass and that common themes have at last emerged concerning the genetics, genes, sequences, molecular biology, genomic distributions, and evolutionary dynamics of drive.

Below, we first describe the expected dynamics of meiotic drive in natural populations. We then survey some exemplar drive systems from plants, fungi, insects, and mammals. These serve as points of reference as we review some of the common genetic and genomic themes to emerge from across systems. Progress on the molecular biology and genomics of drive has revealed extensive, unanticipated interactions among different classes of selfish DNAs, thereby suggesting an extended “ecology of the genome.” Finally, following the success story of TEs, we suggest that the commonalities revealed via forward genetics analyses of meiotic drive justify a pivot to reverse genetics approaches: We now know enough to identify, or at least implicate candidates for, meiotic drive past and present from genome sequence data. We conclude that the consequences of drive for genome evolution are more widespread than generally appreciated.

## Drive dynamics and discovery

To train expectations, we first sketch the possible evolutionary fates of drive mutations. As with any low-frequency mutations, new (low-frequency) drive mutations can be lost by chance. The ultimate fate of drive mutations that escape stochastic loss depends on drive intensity (the degree of transmission bias), drive costs (negative natural selection), and the opportunity for the evolution of drive resistance and/or drive suppression. We distinguish three broad classes of drivers. Some drivers get stuck as balanced polymorphisms, maintained by the opposing forces of drive and negative selection. Some exist as polymorphic but phenotypically masked “cryptic” drivers because drive resistance and/or suppression silenced them before they could go to fixation. Finally, some exist as fixed cryptic drivers that went to fixation but no longer drive either because there are no more drive-susceptible chromosomes (and hence no opportunity for drive) or because they are now suppressed. Cryptic drivers cannot however persist indefinitely; they must innovate to find new ways to drive, e.g. circumvent suppression, or else become pseudogenized by mutation pressure.

These alternative states—polymorphic or fixed, active or cryptic, functional or pseudogenized—figure into how drivers are discovered. Unsurprisingly, the first drivers found also happen to be phenotypically conspicuous: Active polymorphic sex-linked drivers cause distorted progeny sex ratios; the best-known autosomal drivers incidentally distort transmission of linked visible markers; and spore killers cause readily observable cytological phenotypes. As experimental crosses between different strains, populations, or species became common practice, cryptic drivers were discovered: Drivers fixed or suppressed in the genetic background of one population come to be unmasked in the naïve, drive-susceptible background of the other.

Despite the many known cases of drive from across major eukaryotic lineages, we anticipate that drive will turn out to be still more common than generally supposed. To see why, consider how easily drive can go unnoticed: if drive involves unmarked autosomes; if drive is weak; if drive is fixed in the population; or if drive is suppressed. Even deliberate searches via phenotypic screens can be prohibitive, especially in non-model organisms. Together, these considerations highlight ways in which our currently best-known drive systems, i.e. those most readily discoverable, may not be a representative sample. One of the aims of this review is to suggest that the discovery of drive elements and their impacts on evolution can be expanded and accelerated by leveraging the ever-increasing inventory of high-quality genome sequence assemblies and population genomic resources.

## Meiotic drive: some exemplar systems

We next review the genetics, mechanisms, and evolution of four well-characterized systems from plants, insects, mammals, and fungi. We focus on three kinds of drive: female drive, which involves the preferential inclusion of drivers into the single egg, rather than the three polar body cells produced during meiosis; male meiotic drive, which involves the incapacitation of non-carrier spermatids; and spore killer drive, which involves the killing of haploid non-carrier spores. This brief tour of exemplars serves to highlight the diversity and the parallels among female, male, and spore killer drive systems across taxa. In the sections that follow, we will refer back to these (and other) systems.

### *Ab10* female drive in maize

**Figure 1:**
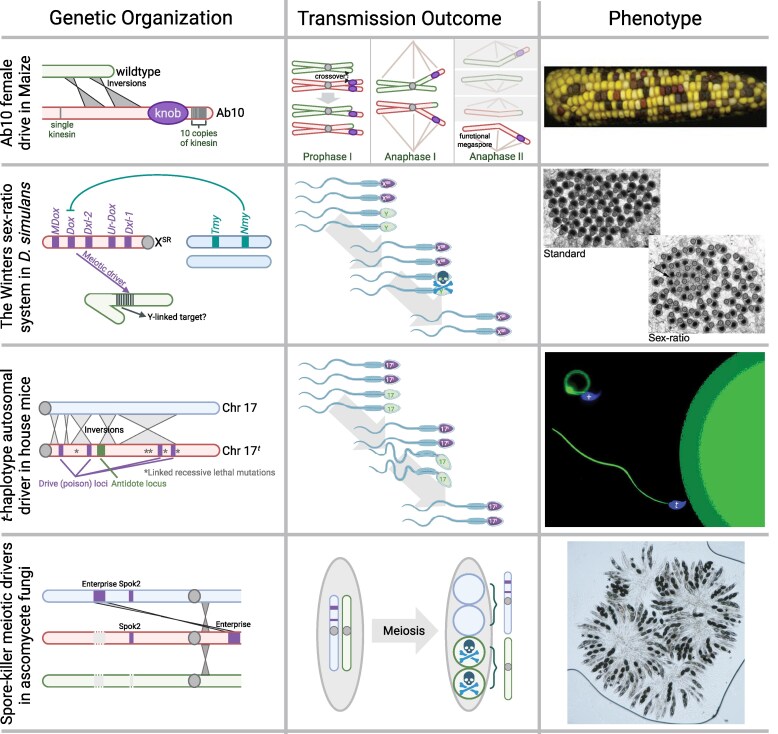
Exemplars of meiotic drive. The organization, transmission, and “phenotype” of four exemplar meiotic drivers for which genetic mechanisms are at least partly characterized: Ab10 female drive in *Maize*, the Winters sex-ratio system in *D. simulans* (image adapted from Tao et al. (2007b) licensed under CC BY 4.0), the *t-haplotype* autosomal driver in house mice, and the *Spore killer* meiotic driver in ascomycete fungi. The yellow kernels contain the Ab10 allele and are overrepresented on the cob. Y-bearing sperm tails in the *D. simulans* Winters system fail to individualize and never mature to become viable sperm. Non-*t*-*haplotype* sperm are malformed and are poor swimmers, so less likely to fertilize the egg (image credit: Alexandra Amaral/MPIMG). Sensitive spores are killed by their siblings, resulting in half the typical number of spores per ascus (two instead of four, marked with an *; image credit: Aaron Vogan). Created in BioRender. Unckless, R. (2026) https://BioRender.com/xvdipa6.

The *abnormal chromosome 10* (*Ab10*) haplotype is a female meiotic drive complex in maize (Fig. 1). Female *Ab10/+* heterozygotes transmit *Ab10* to ∼70% to 83% of progeny (Dawe 2022). Meiotic drive of *Ab10* is countered by reduced seed production and pollen viability when homozygous, so that it persists as a balanced drive polymorphism carried by ∼6% of maize lines (Higgins et al. 2018; Brady and Dawe 2025). The ∼100 Mb *Ab10* haplotype differs from wild-type chr10 by two inversions, several massive tandem repeat arrays (called “knobs”), and expanses of unique sequence (Liu et al. 2020). Two different kinesins encoded on *Ab10* interact with two different forms of knob repeat. These recombine with the wild-type chromosome during prophase I of meiosis. Then, during anaphase II, the kinesins and knobs interact to pull *Ab10* rapidly to spindle poles, preferentially delivering knobbed chromatids to egg cells (Rhoades 1942; Dawe et al. 2018).

### Winters sex ratio male meiotic drive in *Drosophila simulans*

Winters sex ratio is one of three cryptic, polymorphic male meiotic drive system in the fruit fly, *D. simulans* (Fig. 1). Drive is caused by the X-linked gene, *Distorter on the X* (*Dox*), which disrupts the development of Y-bearing spermatids, so that males sire progeny with strongly female-biased sex-ratios (>80%) (Tao et al. 2007a; Tao et al. 2007b). Drive is, however, rarely observed, as an autosomal (*3R*) suppressor, *Not much yang* (*Nmy*), restores balanced X-Y transmission and equal sex ratios (Tao et al. 2007b). *Dox* is a lineage-restricted, chimeric gene that encodes a predicted protamine, a small sperm-specific histone predicted to replace canonical histones to remodel and super-condense spermatid chromatin during spermiogenesis (Muirhead and Presgraves 2021; Vedanayagam et al. 2021). *Nmy* is a retroduplicate copy of *Dox* that expresses predicted hairpin RNAs (hpRNAs) that are processed into endogenous small (∼21 nt) interfering RNAs (esiRNAs) which suppress *Dox*-mediated sex-ratio drive via RNA interference (Tao et al. 2007a; Lin et al. 2018). The Winters system, which comprises *Dox*, *Nmy*, and a presumed (but still unknown) Y-linked target susceptible to *Dox*-mediated disruption, is however part of a large, dispersed family of related putative drivers (*Ur-Dox*, *Dxl-1*, *Dxl-2*) and suppressors (*Tmy*) (Muirhead and Presgraves 2021; Vedanayagam et al. 2021). Across the three closely related species of the *D. simulans* clade (∼250 Ky), ∼18 *Dox-like* (*Dxl*) duplicate gene copies have proliferated within a 1-Mbp interval on the X: 5 *Dxl* genes in *D. simulans*; 12 in *D. mauritiana*; 12 in *D. sechellia*; and, notably, none in any outgroup species. All but one of these *Dxl* duplicates inserted into preexisting “islands” of 359-bp satellite DNAs dispersed throughout the X euchromatin. Last, each of the three species has two autosomal hpRNA genes that produce esiRNAs with predicted targeting to different subsets of *Dxl* genes for silencing (Muirhead and Presgraves 2021; Vedanayagam et al. 2021).

### *t-*haplotype male meiotic drive in house mice

The *t-haplotype* (*t*) is a balanced, polymorphic male meiotic drive complex on chromosome 17 (an autosome) in house mice *Mus musculus* (Fig. 1). In male +/*t* heterozygotes, *t*-sperm enjoy a competitive advantage over *+*-sperm so that 90% to 100% of progeny sired inherit the *t-haplotype* (Silver et al. 1985). Recombination between the *t-haplotype* (*t*) and wild-type (+) chromosome 17 is suppressed under five chromosomal inversions (Swanepoel et al. 2026), resulting in elevated differentiation. The *t-haplotype* has gained gene copy expansions, acquired gene transpositions from other chromosomes, and accumulated recessive lethal and sterile mutations (Lyon 2003; Kelemen and Vicoso 2018; Kelemen et al. 2022; Swanepoel et al. 2026). As a result, drive is balanced by homozygous lethality or sterility (depending on the *t-haplotype* variant), so that the *t-haplotype* is carried by 0% to ∼30% of individuals in a population (Ardlie and Silver 1996; Manser et al. 2011). At least four independently evolved driving loci produce gene products at the early pre-meiotic stages of spermatogenesis, which may allow them to affect the function of both + and *t*-sperm produced by these cells (Bauer et al. 2005, 2007, 2012; Charron et al. 2019). *t* drivers are thought to misregulate their target protein, SMOK (sperm motility kinase), causing abnormal flagellar function (Amaral and Herrmann 2021). The *t*-spermatids counteract this signaling impairment by a non-diffusible insensitive responder, SMOK^Tcr^, which partially restores flagellar function (Veron et al. 2009).

### *Spok* spore killer drive in *Podospora* ascomycete fungi

The *Spok* genes of the model ascomycete fungus, *Podospora*, are single-locus, toxin-antidote spore killers (Fig. 1). In crosses between a strain bearing a specific *Spok* homolog and one without, *Spok* produces a toxin that diffuses to all developing spores and an antidote that remains localized within *Spok*-bearing spores. As a result, *Spok* spores survive, but *Spok*-null spores do not. Across *Podospora* species, *Spok* has diversified into a family of four closely related but functionally distinct variants. *Spok1* is at high frequency in *P. comata* (Vogan et al. 2019). *Spok2* was likely fixed in *P. anserina* in the past, but a derived deletion is now present at low frequency (13.4%) (Vogan et al. 2019; Ament-Velásquez et al. 2022). *Spok3* and *Spok4* occur within a giant transposon named *Enterprise* and can be found in at least four genomic locations in *P. anserina* (Vogan et al. 2021). As individual strains of *P. anserina* possess different combinations of *Spok2*, *Spok3*, and *Spok4*, and these genes have no epistatic interactions, a hierarchy of spore killing phenotypes exists, in which crosses between strains with different *Spok* genotypes will always result in spore killing (Vogan et al. 2019).

## Evolution of genes and sequences involved in drive

In this section, we consider some emerging biological properties and inferred evolutionary dynamics of sequences that function as drivers, drive targets, and drive suppressors.

### Drive origins

New drivers originate as structural and/or genic mutations with the capacity to perturb the mechanics of segregation during meiosis per se or, alternatively, the functionality of spores or haploid sperm that lack the driver. Drive mutations can involve losses of function (*HP1D2^SR^* in *D. simulans*) (Helleu et al. 2016), gains of function via single nucleotide changes at existing genes (*Overdrive* in *D. pseudoobscura*) (Phadnis and Orr 2009), and complex changes that originated de novo or via duplication, truncation (*SD* in *D. melanogaster*) (Larracuente and Presgraves 2012), transposition (*Spok3* and *Spok4*) (Vogan et al. 2021), and/or rearrangement—or some combination of all of these. The asymmetry of female meiosis, for instance, presents opportunities for selfish centromeres, neo-centromeres, and/or selfish alleles at protein-coding genes with functions in chromosome segregation to gain preferential inclusion in the oocyte by, e.g. amplification of existing sequences. The driving *Ab10* haplotype in maize, the *D* locus centromere in *Mimulus*, and the strong centromeres in house mouse comprise expanded blocks of satellite DNAs that recruit proteins that bias spindle attachment/orientation and hence faster polar migration during meiosis I and II, respectively (Finseth et al. 2015; Iwata-Otsubo et al. 2017; Dawe et al. 2018). In contrast to female drive, sperm killers and spore killers tend to involve interactions among protein-coding genes. In killer-target systems, like the *SD* autosomal driver of *D. melanogaster*, a truncated duplicate gene, *Sd-RanGAP*, encodes a trans-acting, diffusible “killer” protein that incapacitates chromosomes bearing sensitive alleles at a cis-acting target locus (see below). In toxin-antidote systems, a diffusible toxin incapacitates competing haploid sperm (*t-haplotype*) or spores (*Spok*) which lack a localized antidote. Drivers can, therefore, arise via simple or complex mutations; drive via loss or gain of function; and/or involve changes in sequence, expression, and/or copy number changes in protein-coding genes or satellite DNAs.

### Driver sequence evolution

A naïve but sensible expectation is that genes and sequences with functions in meiosis and/or gametogenesis, e.g. chromosome segregation, chromatin regulation, and sperm motility, ought to be evolutionarily conserved. The discovery that these classes of genes are instead enriched for histories of rapid evolution and signals of positive selection, e.g. selective sweeps, lineage-specific evolution, recurrent adaptive evolution, and/or rapid structural diversification, therefore demands explanation. In the past, the rapid evolution at “reproduction” genes, broadly defined, was often attributed to sexual selection or sexual conflict (Ferris et al. 1997; Swanson and Vacquier 2002; Clark et al. 2006; Vacquier and Swanson 2011): In mating systems with polyandry (multiply mating females), for example, conflict occurs over control and access to fertilization opportunities. More recently, however, emphasis has shifted to intragenomic conflicts and, in particular, to drive—the competition among haploid segregants within individuals—as a cause for rapid sequence evolution. For new drive mutations, the driving sequence is expected to experience an initial burst of substitutions as it optimizes its efficacy. This innovation phase is analogous to the burst of adaptive substitutions that follows a change in the environment (Gillespie 2004) or to the neofunctionalization of duplicate genes (Ohno 1970). These substitutions might enhance the strength and/or efficacy of drive (e.g. by refining target binding) and/or reduce incidental pleiotropic costs of drive (e.g. reducing off-target effects). For established drivers, driving sequences may experience recurrent bouts of adaptation to counter the evolution of resistance at drive targets and/or drive suppressors elsewhere in the host genome (see below). The potential thus exists for recurrent episodes of iterative adaptation and counter-adaptation during the antagonistic coevolution of drivers, targets, and/or suppressors. These cycles of coevolution can manifest as elevated rates of protein-coding sequence evolution or changes in expression via gene regulatory evolution and/or gene copy number amplification (Kingan et al. 2010; Muirhead and Presgraves 2021; Vedanayagam et al. 2021).

### Targets and antitoxins

In single-locus cases, drive involves competition between alternative alleles, as when strong centromeres prevail over weaker ones (Mimulus *D*), when chromosomes with knobs prevail over those without knobs (maize *Ab10*), or when the driver encodes a toxin-antitoxin gene (*Schizosaccharomyces pombe wtf* system) (Hu et al. 2017; Nuckolls et al. 2017). In multi-locus cases, drive involves the interaction of alternative alleles at two (or more) loci, as in all genetically characterized male drive systems and in some spore killer systems. (Note that the distinction between simple and complex drive is not always clear-cut, as simple drive systems can become elaborated into complex ones, as in the *Ab10*, *SD*, and *t-haplotype* gene complexes; see discussion below.) For these multi-locus cases, it is useful to distinguish cis-acting target sequences versus antitoxin loci that are expressed. The *D. melanogaster SD* system involves a cis-acting target: During spermiogenesis, chromosomes with drive-sensitive alleles at the *Responder* (*Rsp*) locus—long arrays of a satellite DNA—are eliminated due to disrupted chromatin repackaging (Larracuente and Presgraves 2012). In toxin-antidote systems, in contrast, both products are diffusible, but toxin activity is global whereas antitoxin activity is local, so that driver-bearing gametes or spores enjoy differential protection. Some toxin-antidote sequences are dually encoded by a single locus with spatially restricted antidotes. In the *wtf* drive system, all four developing spores are exposed to the Wtf^poison^ protein, while only spores that inherit a compatible Wtf^antidote^ will survive; there, the antidote protein protects by transporting Wtf^poison^ to the vacuoles, leaving +-bearing spores to perish (Nuckolls et al. 2017, 2020; Zheng et al. 2023; Srinivasa et al. 2025). Other toxin-antidote systems are encoded by different, albeit genetically linked, loci with temporally restricted antidotes: In the mouse *t-haplotype*, for instance, the target expresses the antitoxin, SMOK^Tcr^, late during spermatogenesis, limiting its diffusion through cytoplasmic bridges that connect developing spermatids (Veron et al. 2009).

The coevolutionary interaction between drivers and targets is, presumably, escalatory: Drive imposes direct selection against sensitive target alleles, prompting the evolution of target resistance, which in turn prompts innovation by the driver, and so on. As the molecular mechanisms of more drive systems are uncovered, a clearer picture will emerge on the common functional classes of targets and, hence, on the constraining factors in drive-target coevolution. In flies, for instance, the DNA-binding proteins involved in chromatin formation/maintenance/processing that interact with particular cis-acting target sequences during meiosis or spermiogenesis may present general vulnerabilities. Indeed, sex-ratio drivers may be an important but underappreciated factor in the rapid evolutionary turnover of satellite DNA profiles on Y chromosomes (Courret et al. 2023). While toxin-antitoxin systems now present few functional generalities, the temporal or spatial restriction of antitoxin diffusion implies that their regulation (transcriptional and/or posttranscriptional) or biophysical properties (protein size, mobility, activity, or stability) are important.

### Suppressors

In many systems, drive has led to the evolution of second-site suppressors that act, directly or indirectly, to neutralize transmission distortion. Direct suppression can involve, e.g. the transcriptional silencing of drive genes by sequence-specific small interfering RNAs, which can be encoded by paralogs of the drivers themselves. In the case of maize *Ab10*, knob180-mediated drive requires the co-driver *Kindr*, a cluster of kinesins. Interestingly, some wild-type chromosomes carry a cluster of six degenerate, truncated copies of *Kindr* with duplicated-inverted orientations, termed *pseudo-Kindr*, which produce small interfering RNAs that may silence *Kindr* expression (Dawe et al. 2018; Brady et al. 2025). Similarly, in the case of the *Dox* sex-ratio driver of *D. simulans*, the autosomal suppressor, *Nmy*, is a retroduplicate copy of *Dox* with an internal inverted duplication that via hairpin RNA precursors expresses endogenous small interfering RNAs that silence *Dox* (Tao et al. 2007b; Lin et al. 2018). In the *wtf* system, some drive-resistant loci are antitoxin-only genes derived from complete toxin-antidote *wtf* parent genes (Núñez et al. 2018). These cases show that suppressors are not infrequently derived from the drivers themselves, in striking analogy to transposons and their suppressors (Fire 1999; Brennecke et al. 2007). Indirect suppression can, in principle, involve evolution at loci that modulate the pathways by which drive occurs, as predicted for the centromere-specific histone variant (CENP-A, also known as CenH3) modification of centromeric drive (Henikoff et al. 2001; Henikoff and Malik 2002) and strongly suggested in monkeyflowers (Finseth et al. 2021). These driver–suppressor interactions have potential to lead to coevolutionary arms races, with cycles of innovation at driver(s) and counter-innovation at suppressor(s). Curiously, however, there are several systems in which suppressors are expected but, despite extensive surveys, appear absent. No suppressors are known for the *t*-haplotype in mouse (but see Gummere et al. (1986)) or the *Sex Ratio* (*SR*) drive complex in *Drosophila pseudoobscura* despite abundant opportunity, as both appear to be ancient balanced polymorphisms (Price et al. 2019). Why some drivers readily spawn suppressors whereas others do not is unclear.

### Amplification and degeneration

Escalatory conflicts between drivers, targets, and suppressors often entail dramatic changes in copy number for satellite DNAs, protein-coding gene families, and hairpin/small RNA genes. Driving repeats can intensify drive strength via satellite DNA expansions (e.g. maize *Ab10*, *Mimulus D*) that create additional substrate for microtubule attachment, whereas drive targets can evade drive by repeat contraction or elimination (e.g. *Rsp*). Drive genes can intensify drive and/or evade suppression via copy number expansions that, presumably, augment overall expression levels of drivers. Some expansions involve dispersed copies, as in the *Dox* gene family in *D. simulans* and its relatives, whereas others involve tandem amplification, as in the maize Ab10 *Kindr* cluster. Some systems involve co-amplification between drivers and cognate suppressors as in the sex-linked *Slxl1* and *Sly* ampliconic genes of mouse (Cocquet et al. 2012; Kruger et al. 2019). Systems of co-ampliconic gene clusters are enriched on sex chromosomes in *Drosophila* and in mammals, with testis-specific expression, functions in meiosis, RNAi, and spermatogenesis, strongly implicating them as suspected relicts of past drive (Hurst 1992; Ellison and Bachtrog 2019). Notably, the Y-linked counterparts tend to undergo a more extensive amplification, likely owing to the Y chromosome heterochromatic environment and more frequent reliance on error-prone double stranded break repair pathways (Marti and Larracuente 2023). It is important to note that the dynamics of drive do not lead inevitably to long-term, open-ended, perpetual arms races. Should a driver lose its transmission advantage, e.g. by going to fixation, encountering high-frequency resistance, and/or coming under total suppression, it must innovate to reignite drive or else suffer sequence degeneration. Past episodes of drive can thus litter genomes with defunct sequences, a fact that has important implications for functional validation of candidate drivers (see below).

### Phylogenetic distribution of drivers

How particular drivers are distributed among species depends on their dynamics, persistence times, and capacity for movement between species. For the simplest cases, drivers with fast dynamics, e.g. drivers that arise, sweep to fixation, and then degenerate, will tend to be lineage-specific and thus leave lineage-specific genomic changes. Often, however, drivers manage to persist over long time scales in four ways. First, drivers may persist as balanced polymorphisms. Second, drivers may persist via recurrent innovation, which might involve sequence, expression, and/or copy number evolution. Third, drivers may persist by mobilizing to naïve sites in the genome where they can renew drive, as seen in the *wtf* spore killers which have existed in the *Schizosaccharomyces* genus for over 100 million years (De Carvalho et al. 2022) and by the mobile *Spok* elements in *Podospora* (Vogan et al. 2021). Drivers with persistence times longer than typical speciation times can come to be shared among closely related species (*wtf* drivers in fission yeast) by common ancestry. In these cases, the genealogical histories of drivers ought to be concordant with the species history. Other drivers, however, have managed to move between species via gene flow (Meiklejohn et al. 2018; Svedberg et al. 2021).

Drive systems thus have the capacity to influence genome architecture via multiple mechanisms. In the following sections, we summarize ways in which drivers are known or predicted to influence the structure of chromosomes or whole genomes.

## Chromosome evolution

Drivers can exploit preexisting chromosomal contexts, including regions with limited recombination and non-recombining sex chromosomes (see below). But drivers can also remodel their chromosome contexts and thereby trigger larger-scale events in genome evolution, including changes in chromosome structure and number. The kinds of chromosome-scale changes to be expected will depend on whether a particular driver persists as a balanced polymorphism or goes to fixation.

### Chromosome inversions

In heterozygotes, inversions suppress recombination within the rearranged chromosome segment and often cause reduced fertility (fitness underdominance) due to meiotic abnormalities. The establishment of chromosome inversion differences between species has therefore long posed a problem in evolutionary genetics: How can intrinsically deleterious rearrangements spread and, in some instances, go to fixation? Meiotic drive has been suggested as a force to overcome the intrinsic fertility cost in inversion heterozygotes (Hedrick 1981). While drive has not been confirmed as important in the evolution of fixed inversion differences *between* species, it has contributed to the establishment and maintenance of inversion polymorphisms *within* species. The reason is that drivers that persist as balanced polymorphisms are predicted to recruit genetic modifiers that enhance the efficacy of drive (Prout et al. 1973) and suppress recombination (Thomson and Feldman 1974; Charlesworth and Charlesworth 2000). By suppressing recombination, drivers limit losses through the production of weak or non-driving recombinant haplotypes or, worse, “suicide” combinations in which haplotypes with both the driver and sensitive target eliminate themselves (Hartl 1974; Hurst and Pomiankowski 1991). Thus, like other well-known adaptive polymorphisms, e.g. those involved in sex determination, plant mating types, Batesian mimicry, and social strategies (Thompson and Jiggins 2014), polymorphic drivers can evolve into “supergenes,” i.e. multi-locus gene complexes that mediate alternative phenotypes. And like adaptive supergenes, selfish driving supergenes across diverse taxa—female drive in maize, male drive in *Drosophila* and mouse, and spore killers in Neurospora—have converged in the recruitment of chromosome inversions. While recombination suppression confers short-term benefits by tightening linkage among components of multi-locus drivers, it also incurs long-term costs: Reduced recombination is predicted to reduce the efficacy of natural selection. Consistent with this expectation, high-quality genome assemblies of the *Mimulus D*, *SD*, *t*-haplotype, and *Neurospora Spore killer-2* supergenes reveal increased loads (Navarro-Dominguez et al. 2022) of deleterious mutations, including the accumulation of excess repetitive DNAs (Kelemen and Vicoso 2018; Svedberg et al. 2018; Finseth et al. 2022; Navarro-Dominguez et al. 2022; Swanepoel et al. 2026). The deleterious loads of drive supergenes complicate attempts to distinguish direct versus indirect (linked) fitness effects of drive (Bradshaw et al. 2025) and, of course, affect their population dynamics (Lindholm et al. 2016).

### Karyotype evolution

The centromere drive hypothesis provides a compelling explanation for the discovery that centromeric sequences and structures—chromosomal features with conserved functions—and centromere-binding proteins are, paradoxically, among the most rapidly evolving features of genomes (Henikoff et al. 2001; Malik and Henikoff 2001, 2009). Centromere-bound kinetochore proteins interact with the spindle apparatus to mediate chromosome alignment and segregation during meiosis. A “strong” centromere that is better able to recruit, bind, and/or destabilize inner centromere and/or kinetochore proteins might be more likely to secure transmission to the oocyte rather than the polar bodies (Clark and Akera 2021). The rapid evolution of centromere sequence, structure, and organization may well be attributable to recurrent drive via competition for access to oocytes (Henikoff et al. 2001). In principle, driving centromeres could be counteracted by the evolution of novel centromere proteins with reduced affinity to the new centromere repeat (Malik and Henikoff 2001). The centromere drive hypothesis is supported by direct and growing evidence: In mice, driving centromeres are larger, bind more kinetochore proteins, and orient toward egg cells (Pardo-Manuel de Villena and Sapienza 2001; Iwata-Otsubo et al. 2017; Akera et al. 2019); and, in *Mimulus*, the driving *D* haplotype contains a dramatically expanded centromere repeat and rapid evolution of CENP-A/CenH3 (Fishman and Saunders 2008; Finseth et al. 2015).

Centromere drive may be a pervasive factor in karyotype evolution, in three ways. First, selfish centromeric repeat arrays can change rapidly in size, structure, and/or composition. Second, selfish neo-centromeres can invade and displace primary centromere functions, leading to changes in centromere position. Third, centric (Robertsonian) fusions and fissions between chromosomes can alter centromere number, organization, and position. In humans, chickens, and *Drosophila americana*, centric fusions are preferentially transmitted to the egg (Pardo-Manuel de Villena and Sapienza 2001; Stewart et al. 2019; Clark and Akera 2021). In striking contrast, in some laboratory mice, *unfused* chromosomes are preferentially transmitted to the egg (Pardo-Manuel de Villena and Sapienza 2001; Chmatal et al. 2014; Clark and Akera 2021; Boman et al. 2024). The “polarity” of female drive (i.e. whether drive favors fusions versus fissions) likely depends on the direction of asymmetries present in female meiocytes. The spindle apparatus that mediates centromere attachment and segregation can, for example, show asymmetries in the distributions of microtubules emanating from cell poles. Evolutionary reversals in the polarity of the spindle apparatus can thus reverse the polarity of drive, with dramatic consequences for karyotype evolution: Lineages favoring fusions will tend to accumulate metacentric or submetacentric chromosomes, whereas those favoring fissions will accumulate acrocentric chromosomes (Pardo-Manuel de Villena and Sapienza 2001). The centromere drive hypothesis may thus explain the unexpected distributions of chromosome numbers among taxa. Mammal and teleost fish lineages are enriched for extreme karyotypes, with either mostly metacentric or mostly acrocentric chromosomes (Pardo-Manuel de Villena and Sapienza 2001; Molina et al. 2014; Yoshida and Kitano 2021). Closely related mammal species, and indeed populations, can differ radically in karyotype, suggesting frequent reversals of drive polarity are possible (Pardo-Manuel de Villena and Sapienza 2001; Blackmon et al. 2019). While there are instances of female drive in *Drosophila* (*D. americana* (Stewart et al. 2019), *D. testacea* (Keais et al. 2025)), the case for pervasive female drive in karyotype evolution in flies is less clear than in mammals (Bracewell et al. 2019). Finally, even the evolution of holocentric chromosomes, which lack single “monocentromeres,” may represent defensive innovations against centromere drive: Relying on multiple, small, dispersed centromeres distributes microtubule binding along the length of a chromosome, depriving any single centromere of the power to distort transmission (Malik and Henikoff 2009).

### B chromosomes

In addition to standard (“A”) chromosomes, at least ∼15% of eukaryotes (fungi, animals, plants) possess functionally dispensable but stably propagated supernumerary (“B”) chromosomes (Camacho et al. 2000; D'Ambrosio et al. 2017; Oliveira et al. 2024). B chromosomes are thought to derive from standard chromosomes that are rearranged and/or fragmented due to segregation errors and, in some cases, genome instability in species hybrids. B chromosomes persist via a variety of non-Mendelian modes of transmission during pre-meiotic, meiotic, or post-meiotic divisions, leading to their numerical accumulation (Camacho 2022; Houben et al. 2025). These chromosomes are paradigmatic examples of SGEs that exploit meiotic drive mechanisms to increase their transmission rates beyond Mendelian expectations. Such mechanisms include preferential segregation into the egg during female meiosis, mitotic accumulation, or elimination of gametes lacking the B chromosome (Houben 2017; Camacho 2022). Furthermore, songbirds, jawless fish, and some flies carry enigmatic germline-restricted chromosomes. How these chromosomes are maintained remains unclear, but in several instances, meiotic drive has been suggested to play a role (Vontzou et al. 2023). The sequences of B chromosomes are only beginning to be determined but, where information exists, they appear to be enriched for repetitive DNAs (Camacho et al. 2021), protein-coding genes with cell cycle, and chromosome segregation functions (Clark and Akera 2021; Oliveira et al. 2024; Vea et al. 2025).

### Sex chromosomes

Multi-locus male meiotic drivers are overrepresented on sex chromosomes. In *Drosophila*, for instance, more than 20 independent sex-linked drivers have been identified versus a single autosomal driver (Jaenike 2001; Courret et al. 2019). This disparity is at least partly attributable to observational bias: Sex-linked drive distorts progeny sex ratios, an obvious phenotype, whereas autosomal drive easily goes unnoticed. There are however at least two important biological reasons why sex chromosomes are likely hotspots for drive (Hurst and Pomiankowski 1991; Pomiankowski and Hurst 1993; Presgraves 2009; Lindholm et al. 2016; Courret et al. 2019). First, the lack of recombination between X and Y chromosomes precludes the formation of suicide combinations. Second, the extensive sequence differentiation between X and Y chromosomes provides ample substrate for chromosome-specific targets of drive. If satDNAs are common targets of drive (as with *Rsp*), then repeat-rich Y chromosomes may be especially susceptible.

The canonical model of Y chromosome evolution posits that non-recombining, male-limited chromosomes suffer a reduced efficacy of selection and an accumulation of repetitive DNAs (Charlesworth and Charlesworth 2000). It seems doubtful that such relaxed constraints arguments, by themselves, explain the rapid evolution of Y chromosome sequence composition and structure. The rapid evolution of Ys is, however, easy to understand assuming recurrent episodes of X-linked meiotic drive. Y chromosomes might escape drive by, for example, shedding susceptible repetitive target sequences (Lyttle 1989; Courret et al. 2023) and, as a result, sweep to high frequency, thereby reducing nucleotide diversity below standard neutral expectations. In extreme cases, complete loss of the Y chromosome may suppress X-linked drive, so long as the Y is not fertility essential. Interestingly, in *Drosophila affinis*, XO males with a driving X sire excess sons rather than daughters (Voelker 1972). In this system, drive in XY males causes high rates of nondisjunction, production of nullo-Y sperm, and, in consequence, the driver constantly creates resistant O “alleles” (Ma et al. 2022).

Meiotic drive may further contribute to evolutionary turnover of sex chromosomes and transitions in sex determination systems. Theory shows that new sex-determining mutations that arise in proximity to a sex-specific meiotic driver can enjoy transmission advantages (Ubeda et al. 2015), leading to sex chromosome turnover. In some cichlids, for example, a B chromosome that causes female drive acquired a female-determining gene—female carriers produce female-biased progeny by overriding the ancestral XY system (Yoshida et al. 2011; Clark and Kocher 2019). In at least five rodent lineages, driving Y chromosomes are neutralized by feminizing X* chromosomes: Driving Ys produce excess males, but X*Y genotypes develop as females (Saunders and Veyrunes 2021). As a result, these species segregate for the feminizing X* chromosome and the standard mammalian X and Y chromosomes (Saunders and Veyrunes 2021)). Finally, some Y chromosomes may have originated as B chromosomes (Blackmon and Demuth 2015), including in *Drosophila* (Hackstein et al. 1996), Lepidoptera (Lukhtanov and Dantchenko 2002), and planthoppers (Kuznetsova and Aguin-Pombo 2015).

## An extended “ecology of the genome”

The metaphor of an “ecology of the genome was originally invoked to describe interactions among diverse communities of TEs infesting host genomes (Kidwell and Lisch 2000; Brookfield 2005). The proliferation of TEs creates natural parallels with community ecology (Venner et al. 2009; Haig 2024), as TE families compete for finite resources (e.g. chromosomal space, insertion sites, host replicative machinery), evade host defenses (e.g. small RNA-based surveillance systems), diversify to exploit novel host niches (e.g. new insertion target sites, (Brookfield 2005; Venner et al. 2009; Stitzer et al. 2021), and even parasitize one another (e.g. non-autonomous SINEs exploit the protein products of autonomous LINEs) (Kramerov and Vassetzky 2011). Our review of the direct and indirect effects of meiotic drive elements on genome structure, regulation, and evolution suggests that the notion of a “genome ecology” might be profitably expanded. The evolution and consequences of drivers are ultimately determined by their transmission strength, intrinsic costs, and opportunities for innovation and/or persistence. But these features are contingent on a web of intragenomic interactions. Drivers evolve within genomic environments “populated” by other selfish agents—host genes, suppressors, enhancers, other drive systems, TEs, and satellite DNAs—and contingent on intrinsic genomic circumstances—mutation rates, stability, structural variation, recombination rates, chromosome numbers, etc. As in ecology, regulatory feedbacks can develop between these agents and/or between agents and their circumstances.

### Drive–TE interactions

Imagine a SGE that possesses both drive *and* transposition activities: It could drive to high frequency or fixation; and, when fixed and thus faced with possible degeneration, it could transpose to a new genomic position and drive again. This sort of mutually beneficial merger of functions would be an ideal long-term parasitic strategy (Lai and Vogan 2023). In fact, there are cases in which drive and transposition functions have indeed united. In *Podospora*, for example, the *Spok3* and *Spok4* spore killers are embedded within a giant *Starship* DNA transposon, the *Enterprise* (Vogan et al. 2021). *Enterprise* belongs to a large, newly discovered family of transposons (called *Starships)* found across filamentous fungi, and other *Starships* are known that ferry distant homologs of the *Spok* genes, suggesting that this cooperative association may be ancient (Gluck-Thaler et al. 2022). Similarly, in the roundworm, *Caenorhabditis briggsae*, the *msft-1* toxin-antidote element resides within a MULE transposon that allows it to jump within genomes. The toxin *msft-1* itself evolved from a family of proteases associated with Mavericks/Polintons, an ancient, virus-like family of transposons that are horizontally transferred among highly divergent nematode species (Widen et al. 2023). In both cases, active transposable drive elements coexist with inactive degenerate ones, suggesting genomic effects far more pervasive than the current instance. Like the transposable *P*-element in *Drosophila* species, which spread throughout *D. melanogaster* following a horizontal gene transfer in the past 100 years (Kidwell 1983; Daniels et al. 1990) but has relatives throughout animals, transposable drivers may turnover rapidly within species but be essentially immortal at larger phylogenetic scales. These principles may prove important in containing the spread of synthetic drive systems (Box 1).

Box 1. Natural lessons for synthetic drivers Synthetic gene drive systems are designed and built in the laboratory with the purpose of genetically transforming targeted natural populations, especially insect crop pest or disease vector species, on the order of tens of generations (Bier 2022). Synthetic drive gene constructs must encode two functions: drive, to get to high population frequency, and a payload gene, to deliver a desirable phenotype (Champer et al. 2020). Imagine, for instance, transforming populations of *Anopheles* mosquitoes that act as vectors for the *Plasmodium* parasites that cause malaria in humans, limiting their mosquito population sizes, rendering them susceptible to insecticide, transforming females (which transmit *Plasmodium* during bloodmeals) into males (which do not take bloodmeals), or making them altogether incompetent to host the *Plasmodium* life cycle. This project has its roots in the sterile insect techniques from the 1940s (Serebrovsky 1940) and gained steam with Burt's homing drive models (Burt 2003) and the expansion of genetic engineering technologies.

Single gene drivers are attractive from an engineering perspective but may be difficult to manage. To date, much theoretical and empirical work has focused on their short-term population dynamics: How can synthetic drivers be designed to enable controlled spread, to be “resistance-proof,” and to allow managed persistence (Price et al. 2020)? For the present purposes, our questions focus instead on the long-term evolutionary fate of synthetic drivers: Once released, could synthetic drivers acquire novel capacities with downstream consequences for genome evolution? As with natural drivers, synthetic drivers may be liable to spread to related non-target species via gene flow or to transpose to novel chromosomal locations. If synthetic drivers spread but impose fitness costs on carriers, then suppressors are expected to evolve at unlinked loci. Regardless of the genetic design of a synthetic driver, suppressors that use RNAi to silence its payload and/or its drive activities could evolve. If the payload activity is suppressed, then the synthetic drive will lose its intended phenotypic utility. If the drive activity is suppressed, then we might expect counter-adaptations by the driver to escape suppression. Counter-adaptations could involve increases in dosage via duplication or amplification; formation of chimeric sequences by recruitment or fusion with endogenous genomic sequence; and associations with transposons that allow for cycles of renewal via transposition and drive. At some point, synthetic drivers that persist long enough may experience sufficient in situ evolution in natural populations to lose the initially clean distinction between “synthetic” and “natural” drive. If there is a lesson from natural drivers, it is that the long-term trajectories of drive—plausibly even those originating as synthetic constructs—follow certain broad themes but are nevertheless diverse and unpredictable.

The interaction between drivers and transposons can also be less direct. We have already highlighted instances in which suppressors of drive have coopted the host TE surveillance apparatus with, e.g. small RNAs being directed to silence drivers. These specific cases may have broader implications. In particular, if the small RNA surveillance machinery has finite capacity, then directing small RNA-mediated silencing to drivers may entail diminished silencing of TEs (or vice versa): Less drive may come with increased TE activity (or vice versa). In short, host cells may experience tradeoffs in their ability to silence multiple classes of SGEs. Consistent with this prediction, lineage-specific adaptation in the transcription factor, trailblazer, created a novel regulator of two nucleases in the piRNA pathway (AGO3 and aub) (Chen et al. 2025). This innovation in trailblazer is specific to *D. melanogaster*, where *Suppressor of Stellate* (*Su(Ste)*)—a suppressor of a X-linked multicopy gene associated with drive (*Stellate*)—dominates the piRNA pool, suggesting that it evolved in response to a dosage-mediated conflict over drive (Chen et al. 2025).

### Drive–satellite interactions

**Figure 2:**
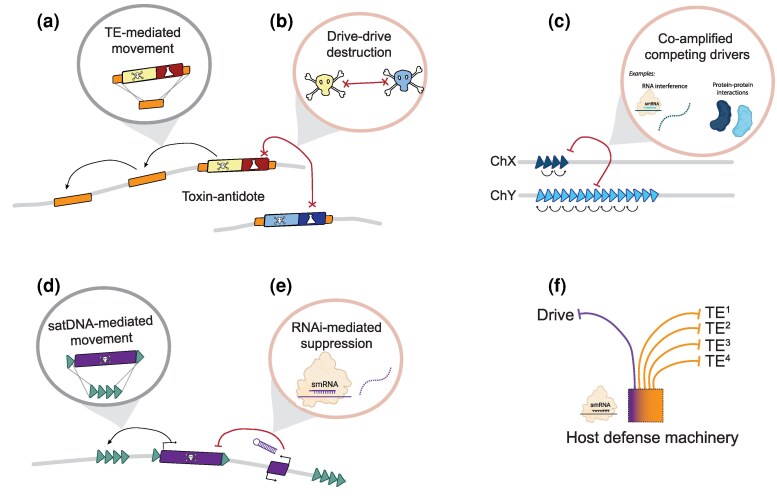
Drive and the ecology of the genome—direct and indirect interactions between SGEs and host cellular machinery. a) Direct interactions between TEs and drivers: In toxin-antidote systems embedded in, or flanked by, TEs, drivers can spread to new genomic locations by taking advantage of the mechanisms TEs use to mobilize, or through recombination-mediated mechanisms (illustrated). b) Interactions also can occur between drive systems: In species with multiple distinct toxin-antidote systems, like the *wtf*s of *S. pombe*, the presence of multiple toxin-producing loci causes the mutual destruction of all gametes. c) Direct interactions between satellite DNAs and drive: In other drive systems, like the sperm-killing *Dox* genes of the Winters system, drivers take advantage of dispersed satellite repeats to spread. These drivers, once flanked by repeats, can use recombination-mediated mechanisms to move to new genomic locations with homologous repeats. d) Indirect interactions between SGEs: The host machinery responsible for silencing TEs (e.g. piRNAs and siRNAs) can be recruited to silence drivers. These small RNA (smRNA)-producing suppressors can emerge from genomic duplications of drivers that produce piRNAs or hairpin RNAs that get processed into siRNAs (illustrated), targeting drive through base complementarity. e) Dosage-sensitive interactions involving sex-linked drivers can lead to arms races that result in gene amplifications. These interactions can be mediated by antagonistic protein–protein interactions between drivers and counter-drivers like the *Slx/Sly* system of *M. musculus* or through RNAi-mediated interactions between X-linked drivers and their piRNA or siRNA-producing Y-linked suppressors (Arlt et al. 2025). f) Because the cellular machinery involved in host silencing have limited abundance, there may be tradeoffs between suppressing drive and TEs. Large increases in the requirement to silence one type of selfish genetic element may titrate away the effectors (e.g. siRNA or piRNA pathway proteins) from responding to another type of selfish genetic element.

Satellite DNAs (satDNAs) are known to have direct roles in drive—as drivers (*Ab10*) or as targets (*Rsp*)—but dispersed blocks of satellite repeats can also have indirect roles by facilitating copy number expansion. Less directly, drivers can increase in copy number, and thus come to occupy new genomic loci, via ectopic exchange among dispersed repetitive satDNAs (Fig. 2). The *Dox-like* meiotic drive gene family members in the *D. simulans* clade species are flanked by multiple copies of the 359-base pair monomer satDNA, which enable their amplification via ectopic exchange (Muirhead and Presgraves 2021; Vedanayagam et al. 2021; Lai and Vogan 2023). Similarly, members of the *wtf* toxin-antidote system were initially named for their association with inactive LTR remnants. These, as well as several other types of repetitive DNAs (e.g. the 5S ribosomal RNA gene), provide substrates for ectopic recombination and structural rearrangements (Nuckolls et al. 2017; Núñez et al. 2018; Eickbush et al. 2019; De Carvalho et al. 2022; Xu et al. 2024). B chromosomes also are often enriched for satellite DNA, but the association with B chromosome meiotic drive and these satellites is unknown. Finally, as with TEs, host regulation of satDNAs relies on RNAi-mediated chromatin regulation and so, if any components of host RNAi machinery are limiting, may entail tradeoffs between the regulation of TEs, satDNAs, and drivers.

### Interactions between drive systems

A single species can host multiple drive systems, with diverse interactions and evolutionary outcomes. If distinct drive systems occupy the same genomic location, they can be in direct competition. In toxin-antidote systems, mutual destruction by alternative killer alleles (Fig. 2) has devastating effects on hybrid fitness in *Neurospora* (*sk-2/sk-3*) (Svedberg et al. 2018) and in *C. tropicalis* (*slow/grow1* and *slow/grow2*) (Ben-David et al. 2021; Pliota et al. 2024). In other cases, if two drivers compete against each other, with neither being fully displaced, long-term arms races may be the norm, prompting the co-amplification of ampliconic gene families (Fig. 2), e.g. on mammal and *Drosophila* sex chromosomes (Ellison and Bachtrog 2019; Bachtrog 2020; Swanepoel and Mueller 2024). Importantly, competition between non-allelic drivers on homologous chromosomes can be resolved if recombination unites them into a single haplotype: In maize, the functionally distinct satellite-kinesin systems (*tr10*-*trkin* and *knob180*-*kindr*) generally co-exist on *Ab10* haplotypes (Brady et al. 2025). Finally, rather than competing, two (or more) drivers can be mutually reinforcing. In the autosomal male *SD* system of *D. melanogaster*, for instance, the strength of *Sd-RanGAP*-mediated drive is boosted by a tightly linked *Enhancer of SD* [*E(SD)*]. However, the discovery that *E(SD)* can drive on its own suggests that this “enhancer” is in fact a co-driver (Larracuente and Presgraves 2012). In an exciting discovery, a selfish X chromosome in *Drosophila testacea* can drive in *both* sexes: The *X*^D^ supergene causes sex-ratio drive in *X*^D^*Y* males (Keais et al. 2017) and female drive in *X*^D^*X* females (Keais et al. 2025). In these cases of mutually reinforced drive, once the first drive allele establishes as a polymorphism, it creates conditions for recruitment of tightly lined coconspirators and suppressors of recombination.

The notion of an “ecology of the genome” was first offered in recognition of modes of TE regulation and interaction. TEs experience limited resources, limited niches, competition, and regulatory feedback. While still early days, our brief survey suggests that an expanded ecology of the genome, one that includes interactions among TEs, satellite DNAs, and drivers within the context of a host's cells and genome, is appropriate and useful. As the genomic natural histories of still more drive systems are characterized, we anticipate that more interactions will be discovered.

## A reverse genetics field guide to drive systems

Analyses of drive systems have almost always proceeded via brute-force forward genetics, going from drive phenotypes to sequences. The critical mass of discoveries achieved in recent years however suggests a new promise for *reverse* genetics, going from sequences to drive phenotypes. To the best of our knowledge, the first instance of a “reverse genetics” anticipation of drive is Hurst's (1992) prediction that *Stellate* (*Ste*) is a relict meiotic driver on the *D. melanogaster X* chromosome. Based on the material reviewed above, the circumstantial evidence implicating *Ste* will seem familiar: *Stellate* (*Ste*) is a testis-expressed, multicopy gene, and its expression is silenced by the Y-linked, multicopy *Suppressor of Stellate* [*Su(Ste)*]; *Ste* and *Su(Ste)* are lineage-restricted, co-ampliconic paralogous sequences; and, notably, both loci are dispensable: Males with *Ste* and *Su(Ste)* are fertile; males with *Ste* but lacking *Su(Ste)* are sterile; and males lacking *both Ste* and *Su(Ste)* are fertile (Chen et al. 2025). Hurst's prescient prediction is now confirmed: *Ste* causes dose-dependent sex-ratio drive (McCormick et al. 2025; Meng and Yamashita 2025), and *Su(Ste)* produces small RNAs that transcriptionally silence *Ste* via RNA interference (Aravin et al. 2001). The *Ste* case shows how identifying genomic vestiges of past conflicts can be the first step in the discovery of drivers rather than the last (Meng and Yamashita 2025).

Moving from forward genetics approaches to reverse genetics, one could accelerate the progress in assessing the prevalence, and hence the importance, of drive as an evolutionary force. The present survey suggests search criteria that might inform genomic scans for candidate drive systems. While no single genomic feature alone is likely to implicate drive, scanning genomes for composite of multiple features—biological functions, genetic properties, and evolutionary signatures—will be powerful.

### Biological functions

Even with the still-shallow information at hand, we can form priors about the biological functions expected to be overrepresented for different kinds of drive. Female drive systems are enriched for centromeres, centromere-proximal satDNAs, centromere-specific histones, kinetochore proteins, and other classes of genes involved in chromosome segregation during meiosis. Male drive systems, in contrast, are enriched for genes involved in meiosis, spermiogenesis, protamines, heterochromatin, nuclear transport, and sperm motility. Spore killer drive systems involve DNA- and RNA-binding proteins, nucleases, and transmembrane proteins. Notably, suppression of female, male, and spore killer drive often involves small RNA-mediated silencing of drivers. In practice, then, small RNA sequence data may be very useful to identify candidate drive loci.

### Genetic properties

We have sufficient information to form priors regarding genetic properties, such as the linkage relationships and structural features of putative drive loci. Killer-target and toxin-antidote systems both involve two functions that may be encoded by separate loci or dually encoded by a single locus (as with some spore killers and pollen killers). Both fulfill the population genetic criterion of a linkage sieve: If the two functions segregate independently, they cannot invade a population. Once established in a population, polymorphic multi-locus drive systems can recruit inversions. The linkage sieve argument also explains the enrichment of multi-locus drive on non-recombining sex chromosomes (Hurst and Pomiankowski 1991; Pomiankowski and Hurst 1993). In addition to linkage relationships, drivers appear to be enriched for exotic structural features: truncated duplications; chimeric genes; duplicated hairpin RNA genes; multicopy genes (dispersed or tandem); co-ampliconic genes; and satellite DNAs. Finally, as many drive loci exist exclusively by virtue of their transmission advantage, they tend to be functionally dispensable for organismal functions.

### Evolutionary signatures

We have sensible priors about expected evolutionary signatures. Short-lived drivers will tend to be lineage-, species-, or population-specific: Novel drivers arise, invade, go to fixation and/or elicit the evolution of suppressors, and then, eventually, degenerate. They live fast but die young. Long-lived drivers—those that escape degeneration—endure via recurring opportunity for drive either by innovation or as balanced polymorphisms. Short-lived drivers and long-lived drivers engaged in cycles of innovation and counter-innovation will be enriched for signatures of selection. For recently active drive systems, the loci involved should be enriched for classic signals of selective sweeps. For drivers and/or suppressors involved in recurrent bouts of innovation, the sequences involved should be enriched for recurrent substitution detectable as elevated *K*_a_/*K*_s_, positive McDonald–Kreitman tests, and lineage-specific accelerations. Balanced drive polymorphisms, often organized as supergenes, should be enriched for strong haplotype structure in the vicinity of the drive loci. Importantly, the population genetic signatures of drive will be indistinguishable from those of organismal adaptation—and vice versa.

### The promise of composite criteria

Integrating biological, genetic, and evolutionary priors has already paid dividends in three genomic “scans” for candidate drive systems. First, building on the profile of the *Ste* case, Bachtrog and colleagues have shown sex chromosomes in *Drosophila* are enriched for lineage-specific, co-ampliconic, testis-expressed genes with biological functions in chromosome segregation and RNAi (Ellison and Bachtrog 2019; Bachtrog 2020). Second, recognizing that protamines appear to be enriched among male drive systems, Chang and colleagues showed that protamine genes are enriched on sex chromosomes and show high rates of turnover (duplication and loss) and histories of rapid protein evolution (Chang et al. 2023). Third, by incapacitating the endogenous RNAi machinery in *D. simulans*, Vedanayagam and colleagues released otherwise quiescent testis-expressed genes that encode DNA-binding domains which happen to be overrepresented on the X chromosome and enriched for lineage-restricted genes (Vedanayagam et al. 2023). As these studies are new, none have functionally confirmed drive in any of the new candidates. But given the combination of biological, genetic, and evolutionary features, it is hard to point to a more compelling model than drive. Functional confirmation seems imminent (see Box 2). Nnote added in revision: Chang  et al. (2025) have experimentally confirmed that rapid evolution of the protamine, *Mst77F*, is indeed attributable to its role in suppressing sex ratio meiotic drive (Park et al. 2023).

Box 2. So you think you have a driver… Suppose you have identified “signatures of drive” at some locus in your genomic data. Your favorite sequence has a history of rapid evolution; a restricted phylogenetic distribution; is duplicated, chimeric, and/or ampliconic; is sex-linked; proximal to an inversion or a centromere; shows germline expression; and encodes RNAi, protamine, chromatin, or chromosome biology function. Now what? While compelling taken together, these kinds of genomic data are still circumstantial.

With the molecular identities of candidate drive alleles in hand, the gold standard is experimental evidence demonstrating drive. Evidence for distorted transmission in a genetic crossing experiment is necessary but not sufficient to demonstrate drive. There are at least three routes to false-positive conclusions. First, even in the absence of drive, apparent transmission ratio distortion can occur by chance; the probability of seemingly non-Mendelian ratios is binomially distributed and depends on the magnitude of the deviation and the number of progeny scored. Second, transmission ratio distortion can occur for reasons other than drive including, e.g. differential viability among genotypes (Fishman and McIntosh 2019). Genotyping gametes or early-stage embryos can mitigate confounding drive with viability effects. Drive and viability are readily separable for sex-specific drivers: Drive is usually sex-specific (see Keais et al. (2025)) but viability effects among progeny (usually) are not. Third, your candidate may be genetically linked to drive without actually causing or contributing to the drive phenotype. This problem is an especially challenging problem for candidate genes in chromosomal inversions. To bolster the case, you could test for associations between natural genetic variation at your favorite locus and the drive phenotype. Naturally occurring loss-of-function alleles or experimentally induced disruptive mutations that ablate drive provide strong evidence. The ultimate proof comes when introduction of a candidate via genetic transformation induces a drive phenotype.

However, even ideal transgenic tests are not fail-safe as there are multiple routes to false-*negative* conclusions. First, a driving transgene may fail to drive if the genetic background is not drive-sensitive or drive-permissive. Second, a driving transgene may fail to drive if the environment, e.g. temperature during development and/or gametogenesis, is not drive-permissive. Third, a “weak” driver can easily induce myriad genomic signatures of drive and yet present a drive phenotype that is effectively undetectable even under well-controlled lab or greenhouse conditions. A driver with a ∼1% transmission advantage can have profound population genetic consequences while also being exceedingly difficult to document under experimental conditions. Finally, some candidate drivers are dead. They caused drive in the past but, after going to fixation or being silenced by other loci, accumulated degenerative mutations.

The fact that drive can be difficult to demonstrate even in well-established model genetic organisms might be daunting. What if your model organism is not amenable to experimental crosses, produces small progeny numbers, and/or lacks the tools for genetic manipulations? Despite these limitations, there are reasons to take heart. Perhaps the necessary genetic tools can be developed, with many approaches to experimental manipulation (e.g. CRISPR) becoming increasingly portable. Perhaps creative proxies for drive are available by, e.g. genotyping gametes, using cytology to score spatial orientation toward eggs rather than polar bodies, etc. While daunting, these challenges are not unique to drive. The challenges of moving from genomic signatures of selection to identifying the genes responsible and providing experimental proof of their adaptive significance are routine for evolutionary biologists.

## Conclusions

Over the past century, drive was discovered serendipitously by close work with a small number of genetic model systems, leaving the impression that drive is an evolutionary curiosity. But as genomes, genomic data, and genetic model systems proliferate, the discovery of new drive systems seems inevitable. We suggest that the discovery of meiotic drive can now be more systematic and deliberate, as eukaryotic genomes are littered with its vestiges. Our review has surveyed the effects of drive on the evolution of gene sequences, expression, and copy numbers; of genes with biological functions in meiosis, chromosome segregation, chromatin regulation, gametogenesis, and RNA interference and of chromosome inversions, karyotypes, and sex chromosomes. And, because drive is by definition non-neutral, its population genomic signatures are often indistinguishable from those of organismal adaptation. While no single feature is sufficient to implicate drive, certain features taken in aggregate can together recommend particular genomic elements for functional interrogation. We therefore believe that we are at an important inflection point in the study of meiotic drive: Rather than rely on circumstance and chance, we now have the means to query its frequency, phylogenetic distribution, and consequences. Our hunch is that, far from a mere curiosity, meiotic drive will become established as an important, if long underappreciated, force in genome evolution.

## Data Availability

There are no new data associated with this article.

## Notes

We also thank the following for their support of individual authors: National Science Foundation Division of Molecular and Cellular Biosciences (NSF MCB CAREER 2047052 to R.L.U.), Division of Environmental Biology (NSF DEB-2344468 to L.F., NSF DEB-1737824 to K.A.D.), National Institute of General Medical Sciences (NIH R35GM119515 to A.M.L., NIH R01GM148442 to D.C.P.), European Research Council (PGErepro to L.R.), Fundação de Amparo à Pesquisa do Estado de São Paulo (FAPESP 2020/06188-5 to A.B.S.M.F.), Royal Society (DHF\R1\180120 to L.R.), Wissenschaftskolleg zu Berlin (support for D.C.P.), and Vetenskapsrådet (Swedish Research Council VR grant number 2021-0429 to A.A.V.).
